# Peer Review of “Awareness, Experiences, and Attitudes Toward Preprints Among Medical Academics: Convergent Mixed Methods Study”

**DOI:** 10.2196/95736

**Published:** 2026-04-17

**Authors:** Kazuki Ide

**Affiliations:** 1 Osaka University, Osaka, Japan

**Keywords:** preprint, medical academics, publishing attitudes, editorial policies, survey

*This is a peer review report for “Awareness, Experiences, and Attitudes Toward Preprints Among Medical Academics: Convergent Mixed Methods Study.”*

## Round 1 Review

### General Comments

This paper [1] reports on a survey exploring the awareness, experiences, and attitudes toward preprints among medical faculty at a university in Türkiye. While the topic may be of some relevance to the academic community, the manuscript contains fundamental issues that must be addressed. As it currently stands, the manuscript is not suitable for publication in the journal. Specific comments are provided below.

### Specific Comments

#### Major Comments

1. Abstract: The authors should specify the name of the university referred to as “a major university in Istanbul.”
2. Abstract: The authors should clarify how the responding editors and the biomedical journals that were manually reviewed were selected.
3. Abstract: The authors are encouraged to present concrete data rather than relying solely on descriptive summaries.
4. Introduction: The authors describe the study as “mixed method,” but it lacks a legitimate integration process between the qualitative and quantitative components. Therefore, the term “mixed method” should be avoided unless such integration is clearly demonstrated.
5. Methods: The authors should specify the name of the university referred to as “a major medical university in Istanbul.” This information is important for assessing the reliability of the study and for confirming ethical approval in a transparent manner.
6. Methods: The authors should indicate the total number of potential participants (ie, the total number of faculty members invited or eligible to participate).
7. Methods: The authors should explain the rationale for dividing the age groups at 40 years.
8. Methods: The authors are encouraged to classify the biomedical journals into basic and clinical categories, in the same way that they categorized the survey respondents, even if some journals may cover both areas.
9. Methods: The authors should provide a list of the journals included in this study.
10. Methods: The section lacks a description of statistical analysis, making the analytical process unclear and only partially reported.
11. Results: The authors should ensure that the findings are presented in alignment with the methods described. As the study does not involve a systematic review but rather a journal policy review, the current framing of the Results section may give a misleading impression.
12. Results: For clarity and coherence, the Results section should be reorganized to reflect the sequence of the study components—for example, starting with the questionnaire survey results, followed by the findings from the editorial and journal policy survey.
13. Results: The authors mention the impact factor, but it is not described in the Methods section. Furthermore, the year and whether it represents the 2-year or 5-year impact factor are not specified.
14. Results: The description of the preprint test, including its content and scoring method, is insufficient, making it difficult to assess its appropriateness.
15. Results: The process for analyzing the qualitative responses is not clearly described, and the presentation of the results is extremely limited.
16. Results: The authors should provide information on how the responses from the editors were summarized. Without this explanation, reviewers and potential readers may find it difficult to interpret the results presented.
17. Discussion: The authors should revise the manuscript for logical consistency and explicitly discuss the limitations of this study prior to submitting it to another journal.

### Minor Comments

1. Tables: The authors should ensure consistent use of commas, periods, and digit formatting. Furthermore, the tables contain typographical errors that need correction.
2. References: The authors should review the reference formatting and ensure that it adheres to the journal’s prescribed style.
